# Impact of Pharmacist-Led Continuous Glucose Monitoring on Clinical Outcomes in People With Type 2 Diabetes in Primary Care: Protocol for a Prospective Cohort Study

**DOI:** 10.2196/67014

**Published:** 2025-05-23

**Authors:** Kevin Cowart, Raechel T White, Kevin Olson, Nicholas W Carris, Karim Hanna, Janice Zgibor

**Affiliations:** 1 Department of Pharmacotherapeutics & Clinical Research Taneja College of Pharmacy University of South Florida Tampa, FL United States; 2 Department of Family Medicine Morsani College of Medicine University of South Florida Tampa, FL United States; 3 College of Public Health University of South Florida Tampa, FL United States; 4 Department of Internal Medicine & Pediatrics Morsani College of Medicine University of South Florida Tampa, FL United States

**Keywords:** type 2 diabetes, continuous glucose monitoring, pharmacist, primary care, family medicine, prospective study

## Abstract

**Background:**

Continuous glucose monitoring (CGM) is increasingly being recognized as the new standard of care for glycemic monitoring in people with type 2 diabetes (T2D). However, despite advances in therapeutics and technology, glycemic control remains suboptimal. Team-based approaches involving pharmacists, particularly in primary care, have shown to be effective in addressing these shortcomings yet have not been rigorously evaluated in the literature.

**Objective:**

Herein we present the protocol for a study that seeks to evaluate the change in hemoglobin A_1c_ (HbA_1c_) in people with T2D using CGM under a pharmacist-led approach as compared with a pharmacist-led approach using no CGM (only self-monitoring blood glucose with a glucometer). We will also assess changes in CGM-derived glycemic outcomes, health behavior, and safety outcomes among the pharmacist-led CGM cohort.

**Methods:**

This is a 12-week prospective cohort study in an academic family medicine department. We will enroll adults with T2D and a HbA_1c_ level of ≥8%. Participants in the intervention cohort will wear a CGM sensor (FreeStyle Libre 2) for 12 weeks and receive structured diabetes self-management education and support from a pharmacist. Each participant in the intervention group will have 5 visits with a pharmacist. The primary objective is the between-group difference in change in HbA_1c_ levels from baseline to 12 weeks between the intervention and historical cohort. Secondary objectives include a change in CGM-derived metrics among the intervention group from baseline to 12 weeks, and a change in health behavior via the Summary of Diabetes Self-Care Activities measure from baseline to 12 weeks in the intervention cohort. A CGM survey will also be administered to participants in the intervention cohort to evaluate changes in diet, physical activity, general lifestyle, and medication adherence. Safety endpoints will also be evaluated. The primary and secondary outcomes will be analyzed within and between groups using descriptive statistics, with a multivariable regression analysis conducted as appropriate to adjust for potential known confounding effects.

**Results:**

This study was funded in July 2023. We began enrolling participants in December 2024. At the time of writing, 3 participants have been enrolled. It is anticipated that we will conclude this study in December 2025 and expect to disseminate results in March 2026.

**Conclusions:**

Results of this study will further elucidate the role of pharmacist-led CGM in primary care.

**Trial Registration:**

ClinicalTrials.gov NCT06572306; https://clinicaltrials.gov/study/NCT06572306

**International Registered Report Identifier (IRRID):**

PRR1-10.2196/67014

## Introduction

More than 38 million adults in the United States have diabetes, with the prevalence estimated to triple by 2060 [1]. The majority of those with diabetes have type 2 diabetes (T2D) [1]. Frequent patient self-monitoring of blood glucose (SMBG) in conjunction with hemoglobin A_1c_ (HbA_1c_) testing is often required in people living with T2D [2]. HbA_1c_ and SMBG have long been recognized as the standard of care for assessment of glycemic control [3]. However, limitations to these metrics exist. HbA_1c_ is an average of glycemia over 2-3 months, with variability in the average glucose range at any HbA_1c_ level [4]. People with similar HbA_1c_ levels can have vastly divergent glycemic patterns [4]. Limitations also extend to SMBG, as it only provides glucose data at one point in time, thereby missing time spent in the normal glycemic range, and potential identification of hyperglycemia or hypoglycemia [4].

Continuous glucose monitoring (CGM) provides glycemic metrics beyond HbA_1c_ and SMBG [5]. CGM is increasingly being recognized as the new standard of care for glycemic monitoring in people with T2D [6-8]. CGM-derived glycemic metrics provide more actionable data for patients and clinicians versus HbA_1c_ and SMBG. Importantly, CGM has also been shown to improve patient engagement and positive health behavior change, improve glycemic control, and reduce hypoglycemia in people with T2D [9-11].

Despite advances in therapeutics and technology, glycemic control remains suboptimal with 50% of patients unable to reach glycemic goals [12]. Failure to reach these goals increases the risk of microvascular and macrovascular diabetes complications [13]. While the reasons for failure to reach glycemic control are multifactorial, clinical inertia has been shown to be associated with an inability to achieve glycemic control [14]. In addition, patient inertia may result from lower self-efficacy and a lack of effective tools to make behavior change, further driving this issue [14,15].

Our recent work suggests that pharmacist-led interventions in close collaboration with medical providers in primary care settings may overcome diabetes-related inertia [16-18]. The trend in team-based care involving a pharmacist is widely recognized at the national and international level and is now the standard of care in a number of medical centers [19]. Several small retrospective studies have found improvements in glycemic control with a pharmacist-led CGM clinical service [20]. However, in our recent scoping review, we identified only 1 published prospective study evaluating a pharmacist-led CGM service [20]. The aim of this work is to further elucidate the impact of pharmacist-led CGM on glycemic control and change in health behavior in people with T2D.

## Methods

### Study Objectives

The primary objective of this study is to determine the impact of pharmacist-led CGM on glycemic control in people with T2D. The secondary objectives are to assess change in CGM-derived glycemic outcomes, change in health behavior, and safety outcomes.

### Practice Setting

This study will take place in the University of South Florida (USF) Health Department of Family Medicine, where people with T2D are routinely managed by pharmacists for chronic disease state management in collaboration with their primary care physician. Routine care consists of appointments that involve reviewing a patient’s blood glucose log or CGM sensor readings, ordering laboratory tests, and adjusting or initiating medication therapy under supervision and in collaboration with medical providers. The study procedures will be incorporated into the patient’s routine appointments. No change in the patient’s routine medical care will occur with their primary care provider or other medical specialists as a result of participating in this study.

### Study Design

This is a 12-week prospective cohort study. Inclusion and exclusion criteria are shown in Textbox 1. Enrolled participants in the intervention group will wear a CGM sensor (Abbott FreeStyle Libre 2; n=20) and receive structured diabetes self-management education and support (DSMES) from a pharmacist in a family medicine clinic. Each participant in the intervention group will have 5 visits with a pharmacist. DSMES will be provided in line with the 2022 National Standards for DSMES [21].

A historical cohort will be used to compare outcomes against the intervention group for the primary outcome. The historical cohort will include participants with T2D and a HbA_1c_ level of ≥8% seen by a pharmacist practicing under a similar scope of practice in a different outpatient clinic within the same health system between January 1, 2020, and January 7, 2024. Participants in this cohort (n=20) will have only used SMBG, and not used CGM within 6 months of the index date (defined as the first visit with a pharmacist during the study time frame). Reporting of research for this cohort study will follow the STROBE (Strengthening the Reporting of Observational studies in Epidemiology) checklist (Multimedia Appendix 1).

**Inclusion and exclusion criteria.**

**Inclusion criteria**

- Type 2 diabetes.
- Hemoglobin A_1c_ level of ≥8%.
- Compatible smartphone with FreeStyle Libre 2 continuous glucose monitoring system.
- Current use and access to a glucometer for self-monitoring of blood glucose.

**Exclusion criteria**

- Any continuous glucose monitor use within 6 months before study enrollment.
- Pregnant and planning to become pregnant during the study time frame.
- History of hypoglycemia requiring third-party assistance.
- History of diabetic ketoacidosis or hyperosmolar hyperglycemic state within 6 months before study enrollment.
- Known allergy to medical-grade adhesives.
- Current use of systemic steroids for any medical condition.
- Current use of dialysis.

### Recruitment

Recruitment began in December 2024, with an expected study completion date of December 2025. Participants included in the intervention group will be recruited from the USF Health Department of Family Medicine. Screening and enrollment will be performed by the pharmacist on the basis of routine care. Participants already scheduled for a visit with a pharmacist for diabetes care and education will be screened for eligibility by review of the medical record. Informed consent will be completed at the first visit.

### Sample Size

The sample size (n=40) was determined on the basis of available research funding, balancing statistical power with financial constraints. While a larger sample could enhance the external validity of findings, budgetary limitations necessitated an approach that maximized efficiency while maintaining sufficient power to detect meaningful effects. This sample size aligns with previous studies of similar scope and ensures the study remains methodologically rigorous within resource constraints [16-18,20].

### Study Visits

An overview of activities that will take place at each study visit for participants enrolled in the intervention cohort is shown in Table 1.

**Table 1:** Study activities.

Study activity	Visit 1 (baseline)	Visit 2 (week 2)	Visit 3 (week 4)	Visit 4 (week 6)	Visit 5 (week 12)
Review eligibility	✓				
Obtain informed consent	✓				
Collect patient baseline characteristics	✓				
Collect vital signs (blood pressure, weight, pulse, and oxygen saturation)	✓	✓	✓	✓	✓
Review and collect medications	✓	✓	✓	✓	✓
Assess diet and fitness activity at baseline	✓				
HbA_1c_^a^ blood sample	✓				✓
FSL2^b^ CGM^c^ sensor placement and education	✓				
Provide 5 FSL2 CGM sensors to participants		✓			
Review ambulatory glucose profile and upload into electronic health record		✓	✓	✓	✓
Administer structured diabetes self-management education and support	✓	✓	✓	✓	✓
Provide food and fitness logs	✓				
Assess and collect food and fitness logs		✓	✓	✓	✓
Administer CGM survey		✓	✓	✓	✓
Administer summary of diabetes self-care activities measure	✓				✓
Assess CGM-related adverse events		✓	✓	✓	✓
Provide after-visit summary to participants	✓	✓	✓	✓	✓

^a^HbA_1c_: hemoglobin A_1c_.

^b^FSL2: FreeStyle Libre 2.

^c^CGM: continuous glucose monitoring.

### Outcomes and Covariates

#### Glycemic Control

To evaluate the primary objective the between-group difference in change in HbA_1c_ levels from baseline to 12 weeks will be compared between the intervention and historical cohort.

#### CGM-Derived Metrics

For the secondary objective, the CGM-derived metrics shown in Textbox 2 will be compared within the intervention group from baseline to 12 weeks.

**Continuous glucose monitoring–derived glycemic metrics.**

- Change in time in range (TIR) at 12 weeks (%).
- Change in mean sensor glucose concentration at 12 weeks (mg/dL).
- Change in time below range at 12 weeks (%).
- Change in time above range at 12 weeks (%).
- Change in glycemic variability (SD and coefficient of variance) at 12 weeks.
- Change in the number of participants increasing by 5% or more in TIR at 12 weeks.
- Change in the number of participants increasing by 10% or more in TIR at 12 weeks.
- Change in the number of participants achieving >70% TIR at 12 weeks.
- Change in the number of participants achieving hemoglobin A_1c_ <7% at 12 weeks.
- Change in the number of participants achieving hemoglobin A_1c_ <8% at 12 weeks.

#### Summary of Diabetes Self-Care Activities

To evaluate change in health behavior we will administer the Summary of Diabetes Self-Care Activities Measure (SDSCA) at baseline and week 12 in the intervention cohort. The within-group difference from baseline to week 12 will be assessed. The SDSCA is a valid and reliable measure of diabetes self-management, which assesses diet, exercise, blood-glucose testing, foot care, and smoking [22]. The strength of the SDSCA includes its brevity and ease of scoring. The SDSCA is a survey scored on an 8-point Likert scale (0-7). Higher scores indicate better compliance with diabetes self-care, whereas a lower score indicates poor self-care performance [22].

#### CGM Survey

We will administer a CGM survey (Table 2) at each visit following the first visit in the intervention cohort. The within-group difference in responses from baseline (second visit) to the end of the study among the intervention group will be evaluated.

**Table 2:** Continuous glucose monitoring survey.

Domain and questions		Response	
**Dietary habits**			
	Have you noticed how different foods affect your glucose levels since using CGM^a^?	Yes No Not sure	
	Have you changed your consumption of sugared beverages since using CGM?	Yes, I have reduced or avoided them No, my consumption is the same I did not consume them before CGM use	
	Have you changed your consumption of high-carbohydrate foods (eg, rice and cereals) since using CGM?	Yes, I have reduced them Yes, I have avoided them No, my consumption is the same I did not consume them before CGM use	
	Do you read nutrition labels more frequently since using CGM?	Yes No I already did this before CGM use	
**Physical activity**			
	Have you increased your physical activity levels since using CGM?	Yes No I was already active before CGM use	
	Are you more likely to engage in physical activity (eg, go for a walk) if you see a rise in your glucose levels?	Yes No Not sure	
**General lifestyle change**			
	Do you feel that using CGM has contributed to a healthier lifestyle overall?	Yes No	
	Since using CGM, how often do you scan to check your glucose levels?	Less than once a day 1-3 times a day 4-6 times a day More than 6 times a day	
	How motivated are you to manage your diabetes since using CGM?	Not motivated Slightly motivated Moderately motivated Highly motivated	
**Medications**			
	Since wearing a CGM, I take my diabetes pills, insulin or both at the right time and as prescribed.	Always Usually Sometimes Rarely Never I already took my medicine at the right time and as prescribed before CGM use	
**Open-ended questions**			
	What specific changes have you made to your diet since using CGM?		—^b^
	What specific changes have you to your physical activity routine since using CGM?		—
	How has CGM affected your overall approach to managing your diabetes?		—

^a^CGM: continuous glucose monitoring.

^b^Not applicable.

#### Safety

Safety endpoints will include an assessment of cutaneous reactions at the site where the CGM sensor is placed. Specific sensor site reactions that will be assessed include erythema, itching, rash, pain, bleeding, bruising, edema, and induration. If any participant experiences a serious cutaneous reaction from the sensor defined as pain, or serious bleeding, they will be withdrawn from the study. If 3 people are withdrawn for that reason, the study will be stopped.

Participants will be screened at the initial visit for any contraindications to using the Abbott Freestyle Libre 2 sensor (including planned magnetic resonance imaging, computed tomography scan, or high-frequency electrical heat (diathermy) treatment during the study time frame.

### Statistical Analyses

Groups will be compared at baseline to determine if any significant differences exist in patient characteristics. The change in the primary and secondary outcomes will be analyzed within and between groups using descriptive statistics (eg, means and SDs for continuous variables such as age, and percentages for categorical variables such as gender). Nonparametric analyses will be used in situations where the variables are not normally distributed. A multivariable regression analysis will be conducted as appropriate to adjust for potential known confounding effects.

The analysis will use an intention-to-treat approach for data analysis. Any participants with missing data or those lost to follow-up will still be included in the analysis. Attempts will be made to match patients on the basis of diabetes medication type and baseline HbA_1c_ in the intervention versus the historical cohorts.

### Ethics Approval

The study protocol was approved by the USF institutional review board (STUDY007451). Informed consent to participate in the study will be obtained from each participant in the intervention cohort. A waiver of informed consent was obtained for participants in the control group as it is not feasible to obtain since participants may no longer establish care at USF. In addition, the electronic health record may have unreliable or inaccurate contact information for the participants, or the participant may be deceased.

All data will be stored in REDCap (Research Electronic Data Capture, Vanderbilt University), a web-based HIPAA (Health Insurance Portability and Accountability Act)-compliant platform hosted at USF that is password protected. All data will be deleted 5 years from when the study has concluded. Confidential data will not be disclosed outside of the study team except as required by law or as allowed by the consent (eg, with the USF institutional review board or those who monitor the study). All data will be deidentified for the analysis. Participants will not receive any compensation for their participation.

## Results

This study was funded in July 2023. We began enrolling participants in December 2024. At the time of writing, 3 participants have been enrolled. It is anticipated that we will conclude this study in December 2025 and expect to disseminate results in March 2026.

## Discussion

### Principal Findings

To our knowledge, this will be the first study to prospectively analyze the impact of a pharmacist-led CGM intervention on glycemia and health behavior in people with T2D in a primary care setting. Our study is unique in that it is not only designed to assess the glycemic benefits of CGM in people with T2D in primary care but also aims to elucidate behavior change resulting from CGM use under a pharmacist-led approach.

We hypothesize that the mean change in HbA_1c_ will be greater for participants enrolled in the intervention cohort as compared to the historical control cohort at 12 weeks. We also hypothesize that participants enrolled in the intervention cohort will experience an improvement in CGM-derived metrics (time in range, mean glucose, time below range, time above range, and glycemic variability) at 12 weeks. For health behavior, we hypothesize that the mean SDSCA score will be higher at 12 weeks compared with baseline. Finally, we hypothesize that an improvement in diet, physical activity, general lifestyle, and medication adherence will be observed on the CGM survey from baseline (second visit) to the end of the study among the intervention group.

### Comparison to Previous Work

Although CGM was first approved for use over 20 years ago, recent technological advancements have made this technology more user-friendly and attainable for a greater number of people living with diabetes, particularly those with T2D. In people with T2D, prospectively driven CGM studies involving pharmacists are currently lacking. In our recent scoping review, we identified only 1 published prospective study evaluating a pharmacist-led CGM service, among a total of 11 published studies in the literature [20,23]. Our methods differ from Fantasia et al [23] in that we are using unblinded CGM devices, which are more commonly used in clinical practice, and are comparing our intervention to a historical cohort managed by a pharmacist in a similar practice setting, rather than to endocrinologists.

In addition, results from our scoping review demonstrated that pharmacist-led CGM was associated with an improvement in glycemic control, with an absolute mean reduction in HbA_1c_ in the literature ranging from –0.4% to –1.8%, which is clinically significant [23-27]. We also found that pharmacist-led CGM was associated with improvements in diabetes self-efficacy [25], improvements in self-care, and improvements in health behavior [28].

### Strengths and Limitations

Several limitations to this study must be mentioned. First, as an observational study design, the lack of randomization may lead to selection bias. For example, it is possible that participants seeking care and who agree to participate in the intervention group might differ in their overall health status and adherence to medical care versus those who do not seek routine medical care. Furthermore, without randomization, differences in baseline characteristics such as medication adherence, lifestyle factors, and socioeconomic status could impact outcomes. Without randomization, it remains difficult to isolate the true effect of the intervention.

In addition, this study may have low external validity, since pharmacist scope of practice varies widely geographically and among health care systems. Further, concerns such as data from a single site and small sample size must also be considered.

On the other hand, several strengths to this study exist, making it a valuable addition to the literature, such as the measurement of clinically significant outcomes (HbA_1c_) as well as the measurement of CGM-derived metrics, which is lacking in many studies. In addition, our study is also using validated measurement tools (ie, SDSCA) to measure health-behavior change. Finally, although observational, the prospective nature of our study establishes a temporal relationship between exposure (to the intervention) and the outcomes, as well as reflecting real-world clinical practice in a primary care setting.

### Future Directions

Our overarching aim is to use the data from this study to support funding to conduct a larger randomized controlled trial that can more rigorously test our hypothesis that pharmacist-led CGM is an intervention that can overcome clinical inertia and improve diabetes-related outcomes in primary care settings.

### Dissemination Plan

The results of this prospective cohort study will be disseminated through presentation in national or international peer-reviewed scientific journals, and at annual meetings of professional conferences.

### Conclusions

There is a need for additional evidence supporting novel approaches to implementing CGM in primary care settings. A prospective cohort study can provide valuable insights into the association of a pharmacist-led approach to implementing CGM.

## Appendix

Multimedia Appendix 1 STROBE (Strengthening the Reporting of Observational studies in Epidemiology) statement and checklist.

Multimedia Appendix 2 Proposal review score sheet for reviewer 1.

Multimedia Appendix 3 Proposal review score sheet for reviewer 2.

Multimedia Appendix 4 Peer-reviewer report from the University of South Florida New Researcher Grant Program.

## Abbreviations

CGM: continuous glucose monitoring
DSMES: diabetes-self management education and support
HbA_1c_: hemoglobin A_1c_
HIPAA: Health Insurance Portability and Accountability Act
REDcap: Research Electronic Data Capture
SDSCA: Summary of Diabetes Self-Care Activities Measure
SMBG: self-monitoring of blood glucose
STROBE: Strengthening the Reporting of Observational studies in Epidemiology
T2D: type 2 diabetes
USF: University of South Florida

## Data Availability

Data are not included in this protocol. The datasets generated and/or analyzed during the current study are not publicly available because this study’s institutional review board does not allow for this study data to be in a public repository. Requests to access the datasets should be addressed to the corresponding author (KC).
