# Supplementary material for: Impact of Pharmacist-Led Continuous Glucose Monitoring on Clinical Outcomes in People With Type 2 Diabetes in Primary Care: Protocol for a Prospective Cohort Study
Source: JMIR Res Protoc. 2025 May 23;14:e67014. doi: 10.2196/67014 (PMC12144473; doi:10.2196/67014)
Supplement: Multimedia Appendix 3 [file resprot_v14i1e67014_app3.pdf]

New Researcher Grant Program - Proposal Review Score Sheet

Principal Investigator: Kelvin Cowart

SCORING:

5.00 = Virtually flawless, negligible weaknesses
4.50 = Extremely strong, a few minor weaknesses
4.00 = Very strong, moderate weaknesses
3.50 = Strong, some major weaknesses that must be addressed
3.00 = Fair, neutral balance of strengths and weaknesses
2.50 = Weak, but with some major strengths
2.00 = Very weak, but with some moderate strengths
1.50 = Extremely weak, with a few minor strengths
1.00 = Virtually without merit, with negligible strengths

Weaknesses:

Minor: an easily addressable weakness that does not substantially lessen impact.

Moderate: a weakness that lessens impact.

Major: a weakness that severely limits impact.

Evaluation Criteria (as communicated in the guidelines)

Potential to contribute to academic unit and applicant's discipline of proposed work.	4.00
Potential to contribute to the applicant's professional development	5.00
Project that is original and innovative in concept and/or approach	4.00
Clarity and soundness of objectives/hypotheses, methods	5.00
Significant publications, exhibitions, performances, writings, especially resulting from prior Internal Awards	4.00
Limited availability of alternative funding for this project	4.00
Budget and Budget Justification	3.00
TOTAL	29.00
OVERALL SCORE:	4.14

Recommend Budget Modification? Comment Below

Need to justify the items listed in Supplies are only for this project.

Comments to Applicant (Strengths and Weaknesses)
