# Supplementary material for: Impact of Pharmacist-Led Continuous Glucose Monitoring on Clinical Outcomes in People With Type 2 Diabetes in Primary Care: Protocol for a Prospective Cohort Study
Source: JMIR Res Protoc. 2025 May 23;14:e67014. doi: 10.2196/67014 (PMC12144473; doi:10.2196/67014)
Supplement: Multimedia Appendix 4 [file resprot_v14i1e67014_app4.pdf]

UNIVERSITY of
SOUTH FLORIDA

University of South Florida | 4202 E Fowler Avenue, | Tampa, FL 33620-4301

Dear Dr. Cowart:

Both Reviewer 1 (score: 3.71) and Reviewer 2 (score: 4.14) met together and reviewed Dr. Cowart's application again.

The innovation of the project is too general. For example, isCGM is a new technology, but the reason that the newly diagnosed T2DM is an important group for this new technology was not explained. With the current presentation, the reviewers could not evaluate whether or not the PI comprehended the scope of background information. The aims presented in the application were unclear and not testable. No research questions corresponded to the aims. The presentation of statistical analyses was not clear (whether or not to answer research questions?). Using bullets to present the study procedure is not acceptable. The potential to secure federal funding (i.e., NIH) from this proposed study is very little. To budget 0.25 FTE for a biostatistics student conducting descriptive statistics, t-test, and chi-square test is not justified well. Both Reviewers agreed that you may consult senior faculty members in the department/college who have successful NIH proposal experience for advices on how to build a stronger proposal.
